# Glutaeal remodelling protocol: Volumization with hyaluronic acid and collagen biostimulation with poly‐L‐lactic acid

**DOI:** 10.1002/ski2.244

**Published:** 2023-05-12

**Authors:** Gladstone Faria, Ricardo Boggio, Marcelo Bellini

**Affiliations:** ^1^ Private Clinic São Paulo Brazil

## Abstract

**Purpose:**

The purpose of this study is to develop an aesthetic treatment protocol for the glutes through the combination of poly‐L‐lactic acid (PLLA) and body hyaluronic acid (HA).

**Patients and methods:**

Six female patients who aimed for glutaeal harmonisation were evaluated. Patients were treated with the combined protocol of Rennova Elleva® (poly‐L‐lactic acid) and Rennova Body Shape® (HA). The results were evaluated through quantitative and ultrasonographic analyses.

**Results:**

The results showed that after 45–90 days after treatment there was an increase in the glutaeal region of the treated patients. The improvement in glutaeal harmonisation was demonstrated through qualitative comparisons and analyses.

**Conclusion:**

Clinical evidence from these six cases suggests that the combined use of PLLA (Rennova Elleva^®^) and HA (Rennova Body Shape^®^) offers an excellent balance between efficacy and safety, with excellent aesthetic results.

## Graphical abstract

The purpose of this study is to develop an aesthetic treatment protocol for the glutes through the combination of PLLA and body hyaluronic acid (HA). The results showed that after 45–90 days after treatment there was an increase in the glutaeal region of the treated patients. The improvement in glutaeal harmonisation was demonstrated through qualitative comparisons and analyses. Clinical evidence from these six cases suggests that the combined use of PLLA (Rennova Elleva^®^) and HA (Rennova Body Shape^®^) offers an excellent balance between efficacy and safety, with excellent aesthetic results.

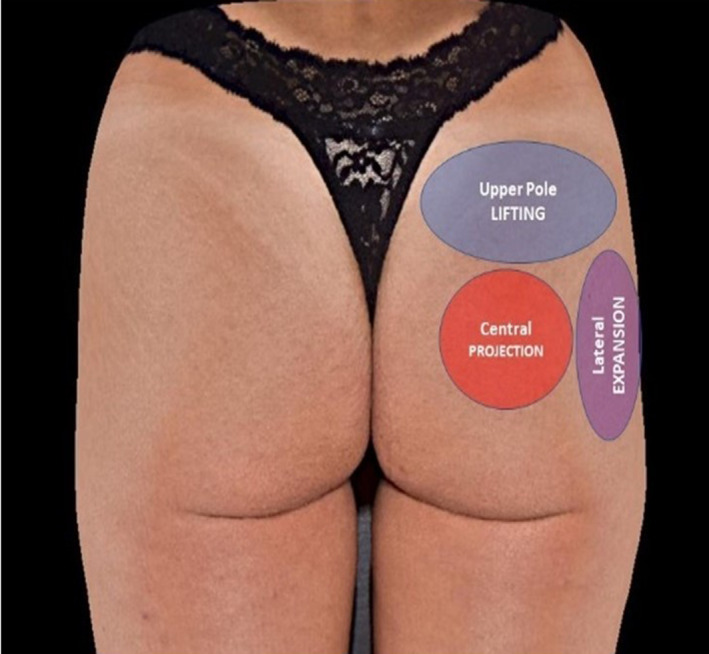

1

**What is already known about this topic?**

- The search for treatment of the glutaeal (or butt) region is growing in the world and the literature is still quite poor with regard to techniques for the beautification of the buttocks. That only one isolated treatment does not give the final results that patients want and, therefore, the creation of protocols involving several techniques is necessary.

**What does this study add?**

- This study demonstrates a global form of butt treatment that involves skin quality and volumization with the use the association of a collagen biostimulator and body hyaluronic acid.

## 2 INTRODUCTION

Aesthetic standards regarding female body contours tend to change according to cultural trends and regional factors. The demand for aesthetic procedures related to glutaeal remodelling is increasing, through the desire for a thin waist, contrasted with a larger hip area, causing many women to seek this type of beauty standard, which is one of the symbols of femininity and involves several aspects such as volume, shape, projection, and quality of the local skin.^1^, ^2^

Body exposure, dissatisfaction with the buttock appearance due to flaccidity (sagginess), depressions, or difference in size between one buttock and the other has increased the demand for fat grafting. This procedure consists of a liposuction surgery performed in different regions of the body, with subsequent reinsertion into the buttocks, requiring hospitalisation, demanding a donor area, and above all, requiring patient downtime, with the possibility of presenting serious complications such as fat embolism and even death.^3^, ^4^, ^5^, ^6^

Due to its permanent characteristic and potential adverse effects, as well as the possibility of injecting products to increase buttock volume, such as silicone implants, and permanent alloplastic materials, such as polymethylmethacrylate, the performance of fat grafting has been questioned.^4^, ^5^

In this context, injectable aesthetic treatments may be a non‐surgical alternative, including collagen biostimulators such as poly‐L‐lactic acid (PLLA) and hyaluronic acid (HA)‐based fillers to improve the contour and increase buttock size.^7^

The use of these products for glutaeal remodelling has gained prominence thanks to the reduced risk of complications, with them being highly sought due to their ability to increase dermal thickness, leading to long‐term volumization, being effective in restoring body volume through its ability to stimulate the production of collagen and elastin, with improvement of cellulite and stretch marks.^5^, ^7^

Injectable dermal fillers are considered safe, indicated for filling medium and deep facial wrinkles, skin depressions, scars, and facial contour definitions. The use of HA for buttock contouring and augmentation is biocompatible and comparable to fillers used in facial treatment, with efficient clinical results.^7^, ^8^

The application of PLLA and HA does not require withdrawal from usual activities, making it a treatment option to achieve an increase in volume. Despite previous descriptions and the high levels of satisfaction related to its use for deformities and increase in the volume of the breasts, as well as the buttocks, the treatment has not yet become popular among the medical community.^8^, ^9^

Glutaeal beauty, in its essence, involves a number of parameters, such as volume, projection, contour, and local skin quality, such as the absence of flaccidity, stretch marks, or cellulite. From this standpoint, the ideal treatment for the glutaeal region should not be isolated; on the contrary, it should involve treating the quality of the local skin and improving its contour/volume, thus employing the association of biostimulators and HA‐based fillers.^7^, ^8^, ^9^, ^10^

Thus, the purpose of this study is to develop an aesthetic treatment protocol for the glutes through the combination of PLLA and body HA, evaluating dermal thickness, glutaeal projection, satisfaction and safety for the remodelling of the body contour.

## 3 MATERIAL AND METHODS

### 3.1 Study design

The study was conducted following all principles of good clinical practice, as well as all ethical protocols, according to the Declaration of Helsinki.^11^ Six female patients, aged 23–43 years, were selected. The cosmetic procedures were performed between January and April 2022. All participants signed the Free and Informed Consent Term and agreed to the publication of their respective clinical cases in a scientific journal.

Inclusion criteria were age greater than 18 years, female, and complaints and expectations consistent with the proposed treatment. Exclusion criteria were age below 18 years with permanent fillers, autoimmune diseases, known allergies to the compounds used, susceptibility to keloids, pregnancy and lactation, bleeding disorders, use of antiplatelet drugs, anticoagulants and thrombolytics, and obesity (body mass index (BMI) >30).

### 3.2 Clinical findings

All patients underwent clinical examination for individual assessment of each case. Several parameters were analysed, including tissue hypotonia (flaccidity), presence of striae, possible points of glutaeal volumization. Possible volumization points were analysed and determined in association with each patient's individual complaint.

### 3.3 Rennova Elleva^®^

Each kit used consisted of 1 vial containing 520 mg (poly‐L‐lactic acid, carboxymethylcellulose, and mannitol). The dilution, homogenisation and application of the product were carried out according to the instructions of the manufacturer of Rennova Elleva^®^ (Ghana R&D CO, LTD – South Korea).^12^ After the completion of the product preparation process, each patient was treated with 20 mL of Rennova Elleva^®^ in the upper and lower quadrant regions of the glutaeus, in both buttocks.

### 3.4 Rennova Body Shape^®^

The Rennova Body Shape^®^ kit consisted of 3 syringes containing 3 mL (cross‐linked HA of non‐animal origin). The preparation of the product was carried out according to the protocol instructions of the manufacturer Rennova Body Shape^®^ (Allanmar International Company SRL, Futerman International Products Argentina).^13^ The product was then applied to the upper quadrant of the right and left glutaeus of the patients participating in the study.

### 3.5 Demarcation of applications

**FIGURE 1:**
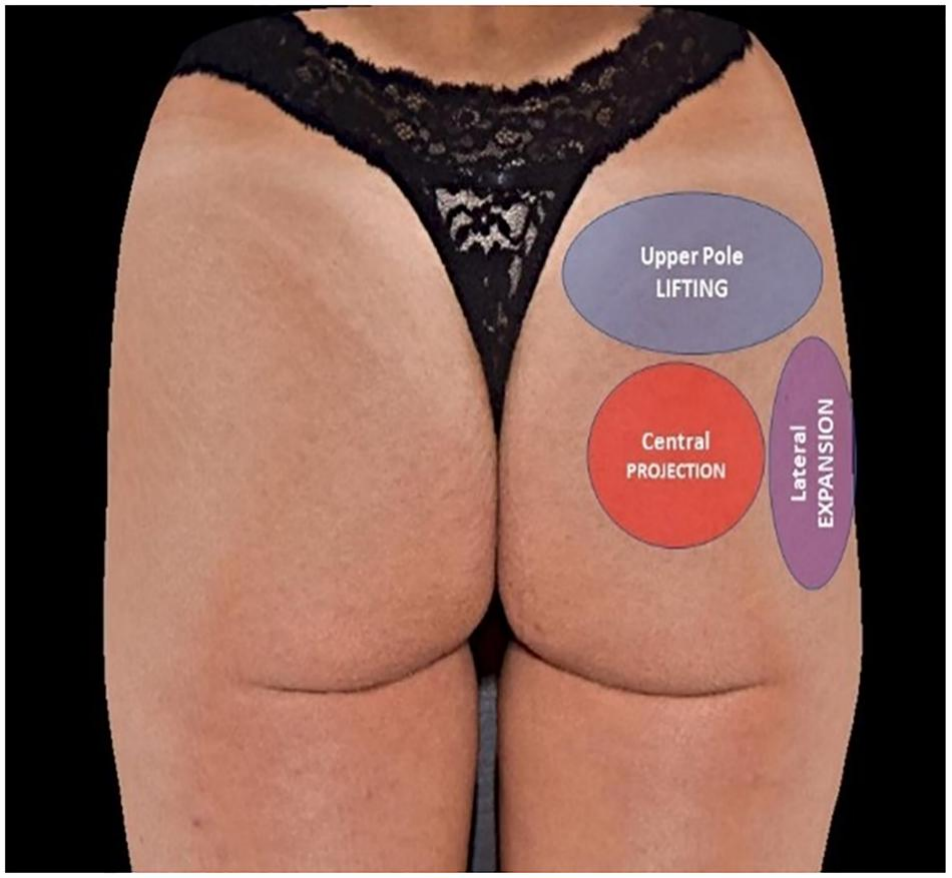
Example of the demarcation performed on the patients. The red colour represents the projection area; the grey colour represents the lifting; and the purple colour represents the expansion area.

Initially, an individual assessment of each patient was performed using the following parameters: lifting (demarcation of the upper pole); expansion (lateral demarcation); and projection (central demarcation) (Figure 1). Fanning technique figures were demarcated to rationalise the technique.

Once the demarcations were finalised, asepsis was performed, followed by application to the buttocks. Applications were performed in the deep subcutaneous plane using the Rennova Cannula^®^ ^14^ (FEEL TECH CO., LTD – SOUTH KOREA) – 18G – 70 mm.

### 3.6 Photographic follow‐up

Photographic records were made prior to the procedure, as well as at 45 and 90 days after the procedure. Study participants had their glutaeal regions photographed in a standardized way using the *QuantifiCare* *®* software programme.^15^ Additionally, using the software, circumference measurements were extracted at various points of the glutaeal region in order to objectively assess whether there was a volume gain in the treated regions.

### 3.7 Ultrasound follow‐up

All patients underwent ultrasound evaluation (Canon Aplio I800 – Canon, Japan), performed immediately after, as well as 45 and 90 days after the procedure. Ultrasound assessments aimed to follow the characteristics of the product over time, anatomical topography, possible migrations, and other complications.

### 3.8 Global aesthetic improvement scale

Satisfaction indices were extracted by applying the Global Aesthetic Improvement Scale (GAIS)^16^ to the aforementioned photographic images in the eyes of the participants, the injecting physician, and a blind evaluator physician (Table 1).

**TABLE 1:** Global aesthetic improvement scale (GAIS).

Degree	Description
A (Very much improved)	(Excellent corrective result)
B (Much improved)	(Marked improvement of the appearance, but not completely optimal)
C (Improved)	(Improvement of the appearance, better when compared with the initial condition, but a touch‐up is advised)
D (Unchanged)	(Appearance substantially remains the same when compared with the original condition)
E (Worsening)	(Appearance has worsened when compared with the original condition)

### 3.9 Follow‐up and outcomes

After the procedure, all patients were screened for possible clinical complications. Assessments were performed immediately after the procedure and new records were performed after 45 and 90 days.

## 4 RESULTS

All six study participants were female, aged between 23 and 43 years, with a mean age of 34 years. The mean BMI was 22 kg/m^2^ (minimum: 19.9 and maximum: 24.1).

### 4.1 Ultrasound assessment

**FIGURE 2:**
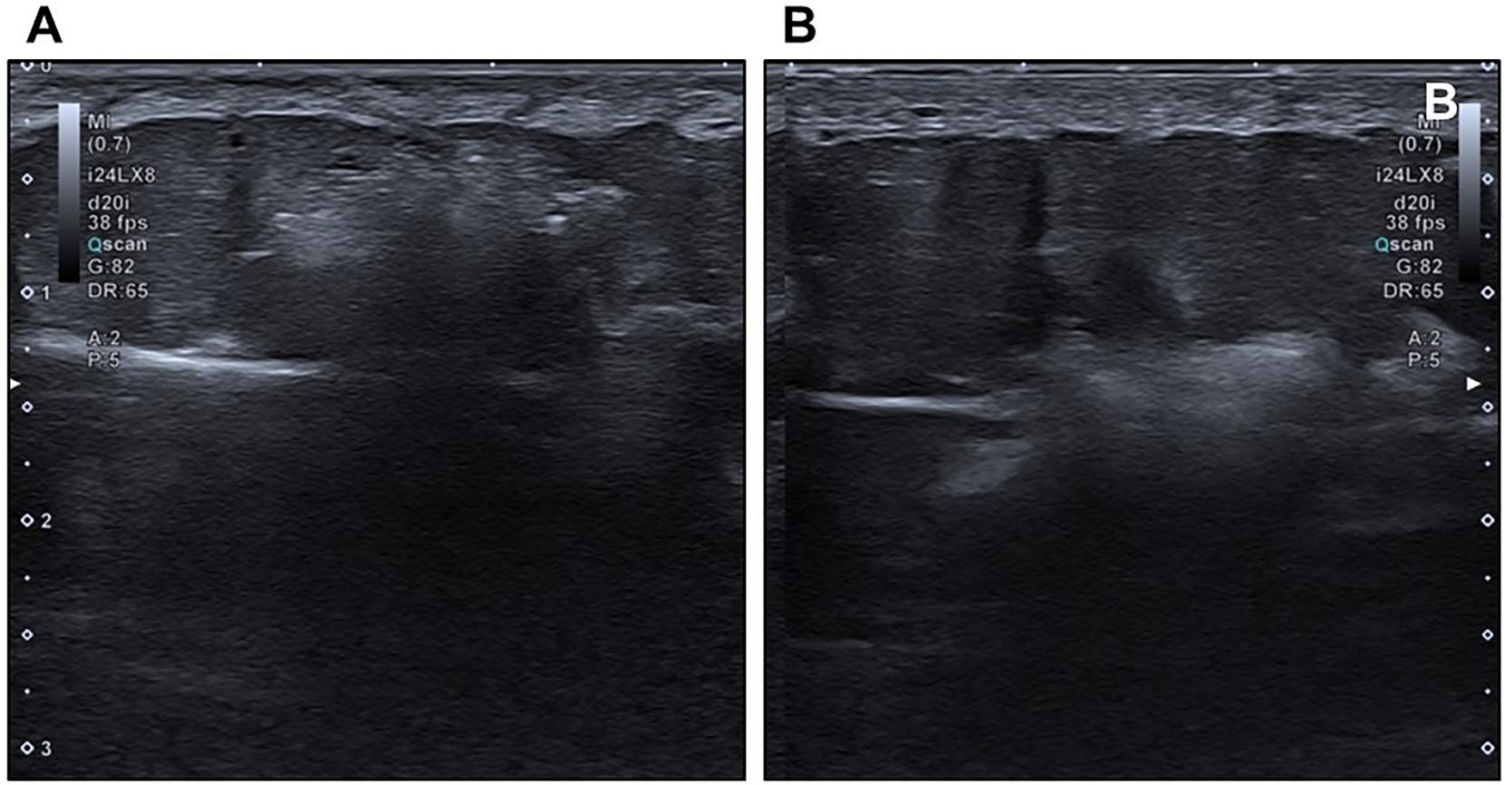
Demonstration of the ultrasonographic aspects of the Rennova Body Shape^®^ body HA. The following were observed: (a) Pure forms of HA; (b) Diluted forms of HA. The images were visualised with the aid of the Canon Aplio I800 device. HA, hyaluronic acid.

The Rennova Body Shape^®^ filler is composed of high‐density HA and was homogeneously located in the subcutaneous tissue of all patients, showing good tissue integration. Its ultrasonographic appearance, in the dilution recommended in this protocol was 1:1. In the absence of dilutions (only focal at one point for assessment purposes), the product appeared as material with a hyperechoic matrix and intermingled hyperechoic foci, with comet tail artefacts and a slight posterior acoustic shadow (Figure 2).

The results showed a volumetric increase in the subcutaneous tissue of the patients in the orthostatic position, more precisely in the glutaeal projection (central region), with a mean difference of 17.3 mm (from 45.8 to 63.1 mm). In the examination performed in the prone position, this volumetric difference was not observed, possibly due to the effect of gravity and tissue spread, as well as the possible compression with the transducer.

**FIGURE 3:**
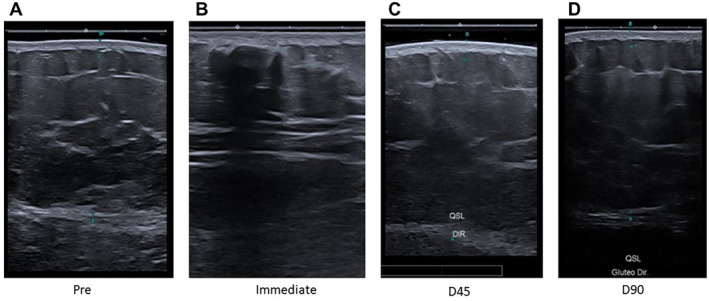
Demonstration of the evolution of ultrasound images of body HA in the glutaeal region. (a) Before the procedure; (b) immediately after application; (c) patient D45; (d) patient D90. HA, hyaluronic acid.

Through the ultrasound procedure, it was possible to identify the product in the subcutaneous tissue layer, with no large‐calibre vessels being observed in this anatomical topography. The follow‐up over the programed time did not demonstrate any migration of the product; on the contrary, in the 45‐day assessment, adequate tissue integration of the product was already observed in all participants (Figure 3).

### 4.2 Quantitative aesthetic assessment

Through the circumference measurements performed using the QuantifiCare^®^ software, the quantitative assessment of the study was performed. The observed results showed that all patients showed gains in their measurements.

Through the evaluation of the GAIS scale, parameters were evaluated in degrees, between A (very much improved) and E (worsening) (Table 1). Added to this, it was demonstrated that there was an overall average gain in centimetres at all points analysed, the same of 1.6 cm, with point A showing the greatest improvement. However, this data still does not represent the entire reality of the treatment, as the lower poles were not treated (Table 2).

**TABLE 2:** Global improvement and improvement in focal points (A – Upper pole; B – Central/projection) (QuantifiCare).

Global improvement	1.6 cm
Point A – Improvement	2.7 cm
Point B – Improvement	1.7 cm

**FIGURE 4:**
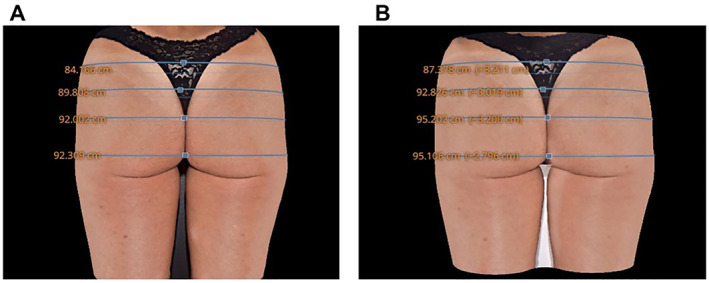
Results statement on patient measurements 4. (a) before treatment. (b) after treatment.

In the assessments of the areas that actually received treatment, that is, either Point A (upper pole of the glutaeus) or Point B (projection area of the glutaeus), the results are even more expressive. For the upper pole, the mean gain was 2.7 cm, while for point B, the gain was 1.7 cm (Figure 4).

**FIGURE 5:**
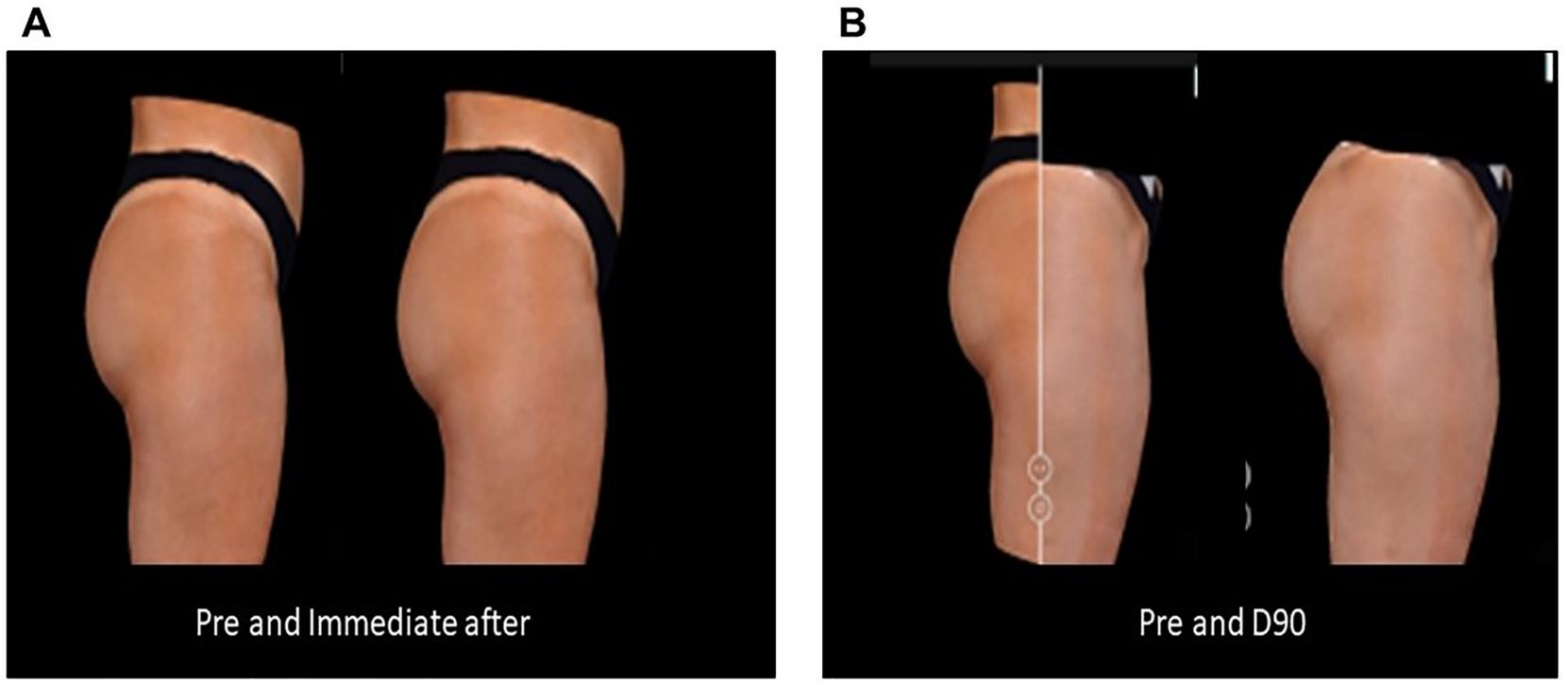
Demonstration of results in patient measurements 5. (a) before treatment. (b) 90 days after treatment.

Results were also tested after 90 days of treatment and the results are shown in Figure 5.

The individualised gains of each patient, at each point, as well as their mean values can be assessed in Table 3.

**TABLE 3:** Measurements in each glutaeal point (QuantifiCare).

Variables	P1	P2	P3	P4	P5
Focal points – Before	x	x	x	x	x
Point A	88.9	90.5	108	84.1	93.1
Point B	97.3	94.5	111.1	89.8	96.1
Point C	99.4	97.6	114.2	92	98.5
Point D	96	100.4	115.4	92.3	97.8
Point E		100.7	115		
**Mean value**	96.65	97.6	114.2	90.9	96.95
Focal points – After					
Point A	90.8	93.2	109.9	87.3	96.9
Point B	98.2	96.2	112.2	92.8	99.5
Point C	100.1	99.2	115.1	95.2	101
Point D	97.6	101.3	116.2	95.1	99.8
Point E		101.2	115.6		
**Mean value**	97.9	99.2	115.1	93.95	99.65
Overall improvement					
Improvement A	1.9	2.7	1.9	3.2	3.8
Improvement B	0.9	1.7	1.1	3	3.4
Improvement C	0.7	1.6	0.9	3.2	2.5
Improvement D	1.6	0.9	0.8	2.8	2
Improvement E		0.5	0.6		
**Mean improvement**	1.3	1.5	1.1	3	2.9

The subjective assessment was carried out with the aid of the injecting physician, through the analysis of photographs and in addition to the application of the GAIS scale. The GAIS scale showed that 100% of the participants had an index greater than or equal to “improved” (4 participants with “improved” and 2 participants with “greatly improved” results). In none of the assessments, either by the professionals or by the participants, were “unchanged” or “worsened” results observed (Table 4).

**TABLE 4:** Comparison between raters based on the Global aesthetic improvement scale (GAIS). *P: participant; A, B, and C: satisfaction levels on the Global aesthetic improvement scale (GAIS).

GAIS	Injector assessment	Blinded	Patients
P1	B	B	C
P2	B	B	C
P3	C	C	A
P4	C	C	B
P5	C	B	A
P6	C	C	C

No complaints were reported by the study participants, nor were any complications diagnosed.

## 5 DISCUSSION

The demand for aesthetic procedures related to the aesthetic improvement of the glutaeal region is increasing, involving a number of aspects such as volume, shape, projection, and quality of the local skin. Although fat‐based treatment is effective in terms of volumization, it has little effect on local skin quality, in addition to being a more invasive procedure, associated with liposuction and requiring anaesthesia, hospitalizations and a recovery period.^2^, ^9^, ^10^, ^17^

Effective procedures, with minimal downtime, which address several nuances related to patients' complaints deserve to be further explored. In this context, a protocol involving collagen biostimulation with improvement of local skin quality, associated with volumization with HA may be the solution to this gap in terms of glutaeal treatment.

The ultrasound assessments of the study demonstrated the safety of the protocol proposed here, as there was total integration of the applied products into the tissues, without reports of adverse effects, such as formation of nodules or foreign body granulomas. Treatments once used for this purpose have been discouraged due to their unpredictability and potential for serious adverse events in the medium and long term.^18^, ^19^

Ultrasonography also contributed to confirming the ability of subcutaneous tissue expansion with the application of HA, with an average gain of 17 mm in terms of projection in the orthostatic position. These results are validated by the measurements performed using the QuantifiCare software. The average gain of measurements was 1.6 cm, but when critically analysed in the regions that are effectively and usually treated (upper pole and projection), the values are even more expressive, with a gain of 2.7 cm for the upper pole and 1.7 for the projection. Treatment with a biostimulator alone does not have a major volumising capacity, even with the application of a high number of vials.^7^, ^20^

From the subjective aesthetic standpoint, all evaluations were considered positive. Even though the same evaluation methodology is applied by the participants, they inevitably correlate their analysis with the improvement observed in their daily lives in terms of skin quality, self‐esteem, firmness, adjustments to clothing and other factors that are not subject to evaluation through photographs. Another factor to be considered was the conservative amount of HA used. Probably, the application of higher volumes would value even more the images of before and after assessment.

Despite the small number of participants, this study was able to demonstrate the safety and efficacy of the protocol involving collagen biostimulation with PLLA Rennova Elleva^®^ and glutaeal volumization with HA Rennova Body Shape^®^. Studies with a greater number of participants, as well as long‐term follow‐up, are important to build a more robust literature in this constantly evolving and growing area of cosmiatry.

## 6 CONCLUSION

The ultrasonographic assessments of the study demonstrated the safety of the proposed study, as there was total integration of the applied products into the tissues, with no reports of adverse effects, such as formation of nodules or foreign body granulomas. Clinical evidence from these six cases suggests that the combined use of PLLA (Rennova Elleva^®^) and HA (Rennova Body Shape^®^) offers an excellent balance between efficacy and safety, with excellent aesthetic results, including glutaeal enlargement and remodelling and high rates of satisfaction of participants and researchers.

## CONFLICT OF INTEREST STATEMENT

None to declare.

## AUTHOR CONTRIBUTION

**Gladstone Faria**: Conceptualisation (equal); Validation (equal); Writing – original draft (lead). **Ricardo  Boggio**: Conceptualisation (equal); Investigation (equal); Validation (equal). **Marcelo  Bellini**: Conceptualisation (equal); Data curation (lead); Formal analysis (equal); Writing – original draft (equal).

## ETHICS STATEMENT

The study was conducted following all principles of good clinical practice, as well as all ethical protocols, according to the Declaration of Helsinki.

## Data Availability

The data that support the findings of this study are available on request from the corresponding author. The data are not publicly available due to privacy or ethical restrictions.
